# The anti-fecundity effect of 5-azacytidine (5-AzaC) on *Schistosoma mansoni* is linked to dis-regulated transcription, translation and stem cell activities

**DOI:** 10.1016/j.ijpddr.2018.03.006

**Published:** 2018-04-01

**Authors:** Kathrin K. Geyer, Sabrina E. Munshi, Martin Vickers, Michael Squance, Toby J. Wilkinson, Daniel Berrar, Cristian Chaparro, Martin T. Swain, Karl F. Hoffmann

**Affiliations:** a Institute of Biological, Environmental and Rural Sciences (IBERS), Edward Llwyd Building, Aberystwyth University, Aberystwyth SY23 3DA, United Kingdom; b Department of Cell and Developmental Biology, John Innes Centre, Norwich Research Park, Norwich NR4 7UH, United Kingdom; c The Roslin Institute, The University of Edinburgh, Easter Bush Campus, Midlothian EH25 9RG, United Kingdom; d Data Science Laboratory, Tokyo Institute of Technology, Tokyo 152-8550, Japan; e University of Perpignan Via Domitia, 58 Avenue Paul Alduy, Bat R, F-66860 Perpignan Cedex, France

**Keywords:** *Schistosoma mansoni*, Epigenetics, 5-Azacytidine, Fecundity, RNA-Seq, Protein synthesis, Stem cells

## Abstract

Uncontrolled host immunological reactions directed against tissue-trapped eggs precipitate a potentially lethal, pathological cascade responsible for schistosomiasis. Blocking schistosome egg production, therefore, presents a strategy for simultaneously reducing immunopathology as well as limiting disease transmission in endemic or emerging areas. We recently demonstrated that the ribonucleoside analogue 5-azacytidine (5-AzaC) inhibited *Schistosoma mansoni* oviposition, egg maturation and ovarian development. While these anti-fecundity effects were associated with a loss of DNA methylation, other molecular processes affected by 5-AzaC were not examined at the time. By comparing the transcriptomes of 5-AzaC-treated females to controls, we provide evidence that this ribonucleoside analogue also modulates other crucial aspects of schistosome egg-laying biology. For example, *S. mansoni* gene products associated with amino acid-, carbohydrate-, fatty acid-, nucleotide- and tricarboxylic acid (TCA)- homeostasis are all dysregulated in 5-AzaC treated females. To validate the metabolic pathway most significantly affected by 5-AzaC, amino acid metabolism, nascent protein synthesis was subsequently quantified in adult schistosomes. Here, 5-AzaC inhibited this process by 68% ±16.7% (SEM) in male- and 81% ±4.8% (SEM) in female-schistosomes. Furthermore, the transcriptome data indicated that adult female stem cells were also affected by 5-AzaC. For instance, 40% of transcripts associated with proliferating schistosome cells were significantly down-regulated by 5-AzaC. This finding correlated with a considerable reduction (95%) in the number of 5-ethynyl-2′-deoxyuridine (EdU) positive cells found in 5-AzaC-treated females. In addition to protein coding genes, the effect that 5-AzaC had on repetitive element expression was also assessed. Here, 46 repeats were found differentially transcribed between 5-AzaC-treated and control females with long terminal repeat (LTR) and DNA transposon classes being amongst the most significant. This study demonstrates that the anti-fecundity activity of 5-AzaC affects more than just DNA methylation in schistosome parasites. Further characterisation of these processes may reveal novel targets for schistosomiasis control.

## Highlights

• The ribonucleoside 5-azacytidine (5-AzaC) inhibits schistosome egg production.
• 5-AzaC modulates schistosome transcription and translation.
• Schistosome stem cell proliferation and maintenance are affected by 5-AzaC.

## Graphical abstract

**Figure:**
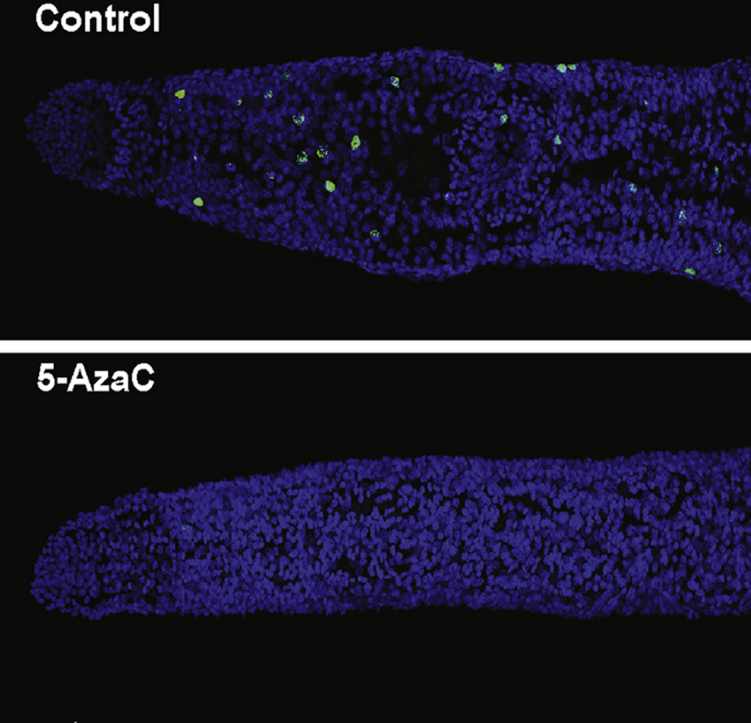

## 1 Introduction

*Schistosoma mansoni* is one of eight species of blood fluke parasite responsible for the neglected tropical disease (NTD) schistosomiasis (Standley et al., 2012). Mostly restricted to sub-Saharan Africa, the Middle East and South America, *S. mansoni* contributes to chronic human pathology and suffering in hundreds of millions of people annually. In *S. mansoni* (as well as all other schistosome species), development is complex and involves free-living stages (snail infective miracidia and human infective cercariae), snail residing forms (asexually reproductive sporocysts giving rise to cercariae) and human dwelling varieties (tissue-migrating schistosomula and sexually reproductive adults). Within the mammalian vascular system, a dioecious state is reached where female schistosomes (paired with males) lay hundreds of eggs every day. These eggs migrate across endothelial barriers into the intestinal lumen and are released together with faeces into a freshwater environment. Upon contact with freshwater, miracidia hatch and actively start seeking a snail host to enable continuation of the lifecycle. However, oviposition also leads to the development of pathological lesions (Warren, 1978), where blood flow away from the site of oviposition (superior mesenteric veins) carries eggs into hepatic tissues. Here, the host‘s immune response to parasite eggs trapped in liver sinusoids results in the development of fibrotic granulomas, portal-systemic shunts, portal vein and pulmonary hypertension, abdominal ascites, oesophageal varices and haematemesis (reviewed in (Hoffmann et al., 2002)). In severe cases, hepatosplenomegaly can also develop as a result of hepatic and splenic vein pressures, reticuloendothelial hyperplasia and hepatic inflammation due to circumoval granulomas. Clearly, strategies that inhibit or significantly decrease schistosome fecundity would simultaneously reduce egg-induced immunopathology as well as decrease schistosomiasis transmission.

Due in large part to transcriptome, proteome, glycome and metabolome analyses, new information directly relevant to sexual maturation, oviposition and egg development have been recently obtained (Fitzpatrick and Hoffmann, 2006; Cass et al., 2007; Hokke et al., 2007; Fitzpatrick et al., 2009; Wu et al., 2009; Ferreira et al., 2014; Pearce and Huang, 2015; Smit et al., 2015; Lu et al., 2016, 2017; Sotillo et al., 2017). Together with anti-schistosome drug discovery efforts, which have identified compounds that negatively affect schistosome oviposition (e.g. (Fitzpatrick et al., 2007; Morel et al., 2014; Edwards et al., 2015; Blohm et al., 2016)), these ‘poly-omics’- related investigations have highlighted genetic targets and molecular pathways underpinning crucial events in schistosome reproduction and fecundity. It is now clear that epigenetic processes also participate in schistosome phenotypic plasticity, egg production and other aspects of the dioecious state (Geyer and Hoffmann, 2012; Carneiro et al., 2014; Cosseau et al., 2016; Picard et al., 2016). While clearly detectable, the significance of cytosine methylation (5 mC) on schistosome developmental processes, particularly in the adult, has been recently debated (Geyer et al., 2011; Raddatz et al., 2013). Studying how cytidine nucleoside analogues, with known inhibitory activity against DNA methyltransferases (DNMTs), affect other aspects of schistosome biology may lead to a more complete picture of the developmentally-important role of this particular epigenetic modification (5 mC).

Two well-known cytidine nucleoside analogues are 5-azacytidine (5-AzaC, a ribonucleoside) and decitabine (DAC, a deoxyribonucleoside). Originally described as cytostatic agents (Sorm et al., 1964), both 5-AzaC and DAC are currently used to treat myelodysplastic syndrome (MDS) and acute myeloid leukemia (AML) (Saba, 2007; Cataldo et al., 2009). Due to its chemical characteristics, DAC only incorporates into newly replicated DNA where it covalently traps DNMTs, effectively inhibiting this enzyme and its DNA methyltransferase activity (reviewed in (Stresemann and Lyko, 2008)). However, in addition to its DNA methyltransferase inhibitory action (Taylor and Jones, 1982), 5-AzaC is additionally known to impede RNA methylation (Lu and Randerath, 1980), decrease protein synthesis (Reichman and Penman, 1973) suppress cholesterol and lipid metabolism (Poirier et al., 2014) and de-repress retroviral expression leading to innate immune response induction (Strick et al., 2016). These pleiotropic activities are likely related to 5-AzaC‘s incorporation into both RNA and DNA pools, which effectively inhibits both DNA- and RNA-methyltransferase function.

In schistosomes, we have previously shown that 5-AzaC, but not DAC, significantly inhibits female specific biological processes including egg production, egg maturation and normal ovarian development (Geyer et al., 2011). While co-cultivation in high concentrations of 5-AzaC did not lead to schistosome death, these drug-associated phenotypes were directly linked to loss of detectable DNA methylation. However, at the time of this previous study, no additional information was obtained to explain how this ribonucleoside affected other aspects of schistosome biology. Here, we continue our 5-AzaC studies and demonstrate that this epigenetic drug (epi-drug) also significantly alters adult female transcription, translation and stem cell activities. Therefore, in addition to DNA hypomethylation, we postulate that 5-AzaC‘s negative effect on schistosome oviposition is multi-faceted and affects core molecular processes (transcription and translation) fundamental to parasite biology and dioecy. These 5-AzaC affected molecular processes are unlikely to be parasite selective, thus precluding the further anthelmintic development of this ribonucleoside analog. However, the downstream pathways affected and the phenotypes modulated by 5-AzaC, as identified in this study, could represent starting points for new chemotherapeutic strategies capable of blocking immunopathology and schistosomiasis transmission.

## 2 Materials and methods

### 2.1 Ethics statement

All procedures performed on mice adhered to the United Kingdom Home Office Animals (Scientific Procedures) Act of 1986 (project license PPL 40/3700) as well as the European Union Animals Directive 2010/63/EU and were approved by Aberystwyth University‘s (AU) Animal Welfare and Ethical Review Body (AWERB).

### 2.2 Parasite material

A Puerto Rican strain (NMRI) of *Schistosoma mansoni* was used throughout the study and passaged between *Mus musculus* (HsdOla:MF1) and *Biomphalaria glabrata* (NMRI albino and pigmented hybrid (Geyer et al., 2017)) hosts. Cercariae were shed from both *B. glabrata* strains by exposure to light in an artificially heated room (26 °C) for 1 h and used to percutaneously infect *M. musculus* (200 cercariae/mouse) (Smithers and Terry, 1965). Adult schistosomes were obtained from *M. musculus* at 7 wk post-infection and used for *in vitro* 5-azacytidine (5-AzaC; Sigma-Aldrich, UK) co-cultures, transcriptome analysis, metabolic labelling experiments and stem cell quantification assays.

### 2.3 Cultivation of adult schistosome pairs for RNA-Seq analysis

Adult male and female schistosome pairs were cultivated in the presence (or absence) of 5-AzaC according to the methodology described in Geyer et al. (2011). Briefly, adult worm pairs were placed into 35 mm petri-dishes (Thermo Fisher, UK) containing DMEM (Sigma, UK) supplemented with 10% (v/v) FCS (Thermo Fisher, UK), 2 mM L-glutamine (Sigma, UK) and 100 mg/ml penicillin/streptomycin (Thermo Fisher, UK). To three replicates (30 schistosome pairs/replicate), 5-AzaC (491 μM in DMEM; final concentration) was subsequently added. An additional three replicates (30 pairs/replicate), lacking 5-AzaC, were included as controls. All six schistosome cultures were incubated at 37 °C for 48 h in a humidified atmosphere containing 5% CO_2_. After 48 h, egg counts were recorded and schistosome worms were subsequently sex-separated and stored in RNAlater (Thermo Fisher, UK) at −80 °C. Worm viability (5 worm pairs/1 ml media in 48 well tissue culture plates; n = 3) was assessed at 7 days post culture initiation (5-AzaC and control treated worms) by WormAssay quantification (Marcellino et al., 2012).

### 2.4 RNA isolation, RNA-Seq library preparation and illumina sequencing

Total RNA was isolated using TRI Reagent (Sigma) and the Direct-zol RNA Mini-Prep Kit (Zymo) according to manufacturer‘s protocol. One μg of total RNA was used per sample for library construction according to the Illumina TruSeq mRNA Library Preparation Kit protocol. Libraries were quantified using Qubit fluorescence spectrophotometry and pooled at equimolar ratios for sequencing in one lane on an HiSeq2500 platform (2 × 101bp format). Reads were demultiplexed, converted to FASTQ format using the bcl2fastq script and assessed for quality control (Andrews, 2010). Raw read files are deposited in the NCBI Sequence Read Archive (SRA) under accession number SRP130864.

### 2.5 RNA-seq read processing, genome mapping and filtering

Analysis of differentially expressed genes (DEGs) was performed as a two-step process. Firstly, Tophat v 2.0.14 was used to perform read mapping against *S. mansoni* genome version v 5.2 with default settings and the genome annotation downloaded from WormBase ParaSite (Howe et al., 2016, 2017). Secondly, transcript assembly was performed using Cufflinks v 2.2.1 to produce a normalised count matrix (genes.count.tables) consisting of 16,234 assembled transcripts, of which 9949 could be mapped to 10,820 unique Smp identifiers (with some transcripts mapping to multiple Smps). Sample replicate quality was assessed by clustering of pairwise Euclidean distances and visualised in a heatmap with treatment (i.e. control or 5-AzaC) as the variable of interest. The biological replicates were, thereafter, combined and differential expression analysis performed using the DESeq2 package (Anders and Huber, 2010) at an FDR of 0.05 (using Benjamini-Hochberg adjustment). Here, 4115 differentially expressed transcripts mapping to 4036 unique Smp gene identifiers were identified at a log2 fold change > ±1. Due to splice variants, 21 of these 4036 Smps were found in both down and up regulated lists of transcripts, giving a total of 4057 differently expressed Smps. Finally, 5102 transcripts were identified by DESeq2 as having non-significant differential expression. All analysis steps were performed with default settings.

### 2.6 Functional enrichment analyses

To investigate functional differences between the 5-AzaC and control samples, the g:GHOSt function (moderate filtering) within the g:Profiler webtool was used for the gene ontology (GO) enrichment of DEGs (Reimand et al., 2016). For the identification of pathways associated with each set of genes, *S. mansoni* pathway data were downloaded from BioCyc (Caspi et al., 2016) and, thereafter, visualised using the Neo4j graphical database (Webber, 2012). The BioCyc data consist of a series of flat files, from which data on genes, proteins, reactions, pathways and compounds were extracted. These were then stored as nodes in a Neo4j graph, with the relationships between them represented as edges. The edges included 1074 enzyme reactions (linking reaction and protein nodes) with the proteins encoded by genes and the pathway hierarchy. The transcriptome expression data, stored as log2 fold change, for each gene, were loaded onto this graph as another set of nodes with edges created to link the data to the respective gene node. Utilising the cypher language that is part of the Neo4j system, the network was searched for reactions and the metabolic pathways to which the enzymes belonged were subsequently defined: 137 and 34 differentially expressed enzymes were identified with minimum log2 fold changes of at least ±0 and ± 1, respectively. In the set of 137 enzymes, there were 94 unique Smp gene identifiers (some enzymes were found in multiple pathways); similarly, there were 23 unique Smps in the set of 34 enzymes. The enzyme network with a log2 fold change > ±1 was subsequently converted into a Venn diagram for visualisation purposes. To illustrate an overview of the genome locations and differential expression levels of the 4057 Smps (4115 transcripts) in the filtered (log2 fold change > ±1) RNA-Seq dataset, a Circos plot (Krzywinski et al., 2009) was generated using the *S. mansoni* genome v 5.2. Further characterisation of DEGs was performed by extracting KEGG BRITE (Kanehisa, 2016) functional hierarchical information for each mapped Smp ID (genome v5.2). Over-representation analysis was carried out on differentially expressed Smps identified by DESeq2. Significantly over-represented pathways (*p* < *0.05*) were identified and plotted using clusterProfiler (Yu et al., 2012) in R. Finally, a heatmap was used to visualise transcripts that were significantly differentially expressed (adjusted *p*-value < 0.05) as a response to 5-AzaC. Transcripts were ranked based on increasing values of their log2 fold change. The number of stem cell associated transcripts (Collins et al., 2013) found in these DEGs were then displayed on this heatmap.

### 2.7 Significantly differentially expressed repeats

The differential expression of repeats was performed using RepEnrich (Criscione et al., 2014). This software handles the technical difficulties that arise when mapping reads to repetitive locations in the genome (reads deriving from repeats rarely map to unique genome locations). Following the recommended protocol, we first mapped the reads uniquely to the genome using bowtie (with the –m 1 option), before calling the RepEnrich.py script with an existing annotation of repeats in bedfile format (Lepesant et al., 2012) to obtain the counts for each repeat subfamily in each of the samples. Subsequently, the three control and three 5-AzaC treated samples were assembled into a 2814 × 6 matrix, where the rows correspond to the 2814 repeat subfamilies and the columns correspond to the 6 samples (3 control vs. 3 treatment samples). The repeat subfamily counts in each control and treatment sample were normalised to account for sequencing depth. Of the 2814 repeat subfamilies, those with 0 counts in treatment or control making a log fold-change impossible to calculate were excluded from further consideration (726 repeat subfamilies in total). All analyses were carried out in R.

To identify interesting candidate repeat subfamilies for further analysis, we used a filtering approach consisting of two steps. First, we analysed the normalised data with Welch's *t*-test sequentially to derive a *p*-value for the null hypothesis of no differential expression between control and treatment samples. At this step, no adjustments for multiple testing were applied. For each repeat subfamily *i*, we also calculated its log fold change (*LFC*_*i*_) as follows:

$$LFC_{i}={\text{log}}_{2}\left(\frac{m_{c}}{m_{t}}\right)$$

where *m*_*c*_ is the mean normalised value of the control samples of the repeat subfamily *i*, and *m*_*t*_ is the mean normalised value of the treatment samples of the repeat *i*. Given the problems with interpretation of data with “small fold change, small variance” and “large fold change, large variance”, we combined the *p*-value and the *LFC*_*i*_ into a single ranking score as follows (Xiao et al., 2014):

$${\text{score}}_{i}=-{\text{log}}_{10}\left(\text{p}\;\text{value}\right)\times LFC_{i}$$

Here, a large negative or positive score indicates that the corresponding repeat subfamily is overexpressed in the treatment or control samples, respectively. To assess the significance of the ranking scores for the remaining 2088 repeat subfamilies, we used a random permutation test as the second step in the filtering process, which addresses the problem of multiple testing. Briefly, we randomly permuted the normalised counts per column 10,000 times and then re-calculated the resulting scores for each repeat subfamily *i*. The distribution of the permutation scores reflects the empirical distribution of the score under the null hypothesis of no association between the counts in the samples and treatment. Then, for each repeat subfamily, we checked how many permutation scores are more extreme than the unpermutated score. The fraction of these scores is the permutation *p*-value for score_*i*_. We included only those repeat subfamilies for which the random permutation testing yielded a significant permutation *p*-value (<0.05).

### 2.8 Metabolic labelling of adult schistosomes and solubilisation of proteins

Adult schistosomes were sex-separated and transferred into 48-well tissue culture plates at a density of 20 worms per well. Wells contained 1 ml of DMEM minus Met (methionine) with and without 5-AzaC (491 μM). Control wells for each gender, containing DMEM plus Met (200 μM final concentration), were also included. The parasites were incubated at 37 °C in 5% CO_2_ for 4 h, to allow depletion of methionine reserves of parasites cultured in DMEM minus Met. The labelled methionine analogue Click-iT AHA (L-azidohomoalanine, 20 mM in DMSO) (Life Technologies) was then added to DMEM – Met cultures to give a final concentration of 200 μM. After 24 h, a 70% media exchange was performed. After 48hr, worms were removed from culture and washed 3 × in 1 X PBS to eliminate all residual media components.

Worm samples were subsequently suspended in 50 μl lysis buffer (50 mM Tris-HCl + protease inhibitors (cOmplete, mini, EDTA-free tablets, Roche), pH 8.0) and solubilised using a Qiagen Tissue Lyser LT and 7 mm stainless steel beads at 50 Hz. Solubilisation was performed for a total of 3 min and included 4 × 30 s bursts with a 30 s incubation on ice between cycles. Samples were then placed in a sonicating water bath for 3 × 30 s (30 s incubation on ice between cycles) to shear the DNA. Samples were centrifuged at 18,000 × *g*, 4 °C for 20 min to pellet cell debris, haemozoin (Truscott et al., 2013) and insoluble protein aggregates. The supernatant was removed and processed for detection of metabolic label.

### 2.9 Detection of metabolic label, SDS-PAGE, western blotting and quantification of nascent protein synthesis

The Click-iT detection reaction was performed on whole protein samples (50 μl) using Biotin Alkyne (Life Technologies, UK) and the Click-iT Protein Reaction Buffer Kit (Life Technologies, UK) according to the manufacturer‘s instruction. After the detection reaction, each protein sample was precipitated by adding 600 μl methanol, 150 μl chloroform and then 400 μl ultrapure water in that order; brief vortexing was implemented after each addition. Samples were then centrifuged at 13, 000–18, 000 × *g* at RT for 5 min before carefully removing and discarding the upper aqueous solution. A further 450 μl methanol was added to the samples, which were again mixed by brief vortexing before repeat centrifuging at 13, 000–18, 000 × *g* to pellet the precipitated protein. The supernatant was discarded and the methanol wash (450 μl) was repeated twice more. Finally, all methanol was removed and the protein samples were dried by vacuum centrifugation and resolubilised in 80 μl SDS-PAGE sample buffer (8 M urea, 2% CHAPS (3-3-cholamidopropyl-dimethylammonio-1-propanesulphonate), 33 mM DTT (dithiothreitol), bromophenol blue).

Protein samples were separated by 1 dimensional SDS-PAGE (precast 4–12% acrylamide gradient gels, Life Technologies, UK). For male worms, 5.0 μg of protein sample was loaded per lane and for female samples, 2.5 μg was used. Gels were electrophoresed according to the manufacturer‘s instructions and were either Colloidal coomassie stained (Majek et al., 2013) or subjected to western blot analysis (Hoffmann and Strand, 1997) using 1: 1000 diluted streptavidin-horseradish peroxidase (HRP) polymer (ultrasensitive, Sigma-Aldrich, UK) as the primary antibody and visualised using enhanced chemiluminescence (ECL Plus, GE Healthcare).

ImageJ was used for quantitative analysis of nascent protein synthesis using three separate western blot images/treatment. The total pixel intensity (derived from fluorescence produced by streptavidin-HRP labelling of biotin tagged proteins) was measured individually for male and female protein extracts in ImageJ. Lanes were manually annotated for each sample and the total pixel intensity was measured within this region. The pixel intensity measured for male and female negative controls that contained no AHA (DMEM plus Met only) were used as a measure of background. These were subtracted from the associated total pixel intensity values for AHA and AHA+5-AzaC extracts. The average (+/− standard error) percent AHA incorporation in 5-AzaC treated male and female schistosomes (normalised to AHA treated worms only) was subsequently calculated.

### 2.10 Quantification of stem cell populations in adult worms

*In vitro* 5′-ethynyl-2′-deoxyuridine (EdU) labelling was performed as previously described (Collins et al., 2013). Briefly, 5-AzaC treated (491 μM) or control adult females were cultured for 48 h then pulsed with 10 μM EdU and cultured for a subsequent 24 h. After 72 h, female schistosomes were fixed, stained and prepared for laser scanning confocal microscopy (LSCM) imaging as previously described (Collins et al., 2013). Anterior regions were imaged and used to determine the relative number of EdU-labelled nuclei found in 5-AzaC treated worms compared to those found in control groups. For this quantification, LSCM images were acquired using a Leica TCS SP5II confocal microscope using the 40X lens (NA 1.25), accruing a total of 60 sections for each Z-stack. For each Z-stack, the fluorescent intensity of the DAPI and EdU channels (405 nm and 488 nm respectively) were used to calculate the total volume (μm^3^) occupied by each fluorophore using the Surface tool in Imaris v 8.2 (Bitplane). The percentage of EdU positive nuclei was calculated by dividing the volume of the EdU channel by the volume of the DAPI channel. To investigate significant differences between the treatments, a student‘s *t*-test was performed.

## 3 Results and discussion

**Fig. 1:**
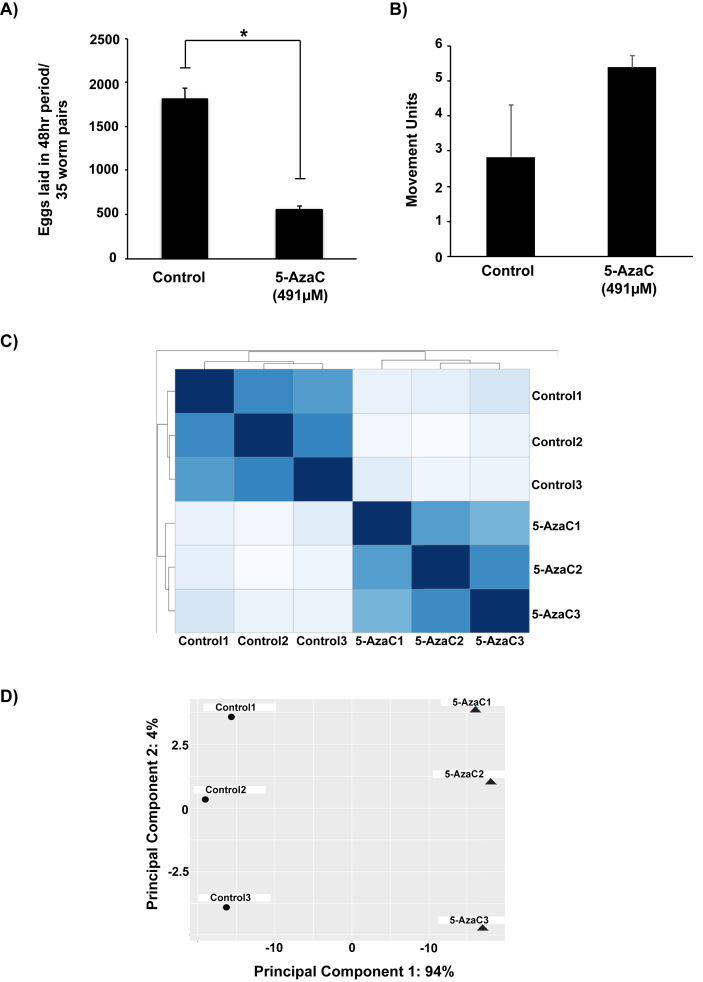
**The anti-fecundity action of 5-AzaC modulates adult female transcriptomes. A) 5-AzaC significantly inhibits *S. mansoni* egg production**. Thirty-five adult worm pairs were cultured either in the presence or absence of 491 μM 5-AzaC. Each culture condition was replicated (n = 4) and eggs were collected from each culture well after 48 h and counted using a sedgewick rafter. Error bars represent the standard error of the mean (SEM) and a two-tailed *t-*test was performed. * corresponds to *p* < 0.05. **B) 5-AzaC does not affect adult schistosome viability.** Five worm pairs were cultivated in the presence (n = 3) or absence (n = 3) of 491 μM 5-AzaC for 7 days. Quantification of worm movement (see video in Supplementary Fig. 1) was performed using WormAssay (Marcellino et al., 2012). **C) Data quality visualisation of RNA-Seq dataset.** The heat map illustrates the Euclidean distance measurements amongst RNA-Seq replicates calculated from the variance stabilising transformation of the count data using the DESeq2 package and provides an overview of similarities/dissimilarities amongst biological replicates (n = 3 for each treatment). Colour shades indicate level of similarity between libraries. **D) Clustering of biological replicates assessed by principal component analysis (PCA).** A PCA was computed using the DESeq2 package in R. Both treatments are represented by three biological replicates (n = 3), which are separated by their first two principal components (percentages of total variation accounted for by the PCs are show in parentheses). The control samples are represented by circles and the 5-AzaC samples by triangles respectively. (For interpretation of the references to colour in this figure legend, the reader is referred to the Web version of this article.)

The ribonucleoside analogue 5-AzaC is a known inhibitor of DNA methyltransferase activity and, thus, has been used to treat epigenetic disorders associated with DNA methylation dysregulation such as myelodysplastic syndrome (MDS) and acute myeloid leukemia (AML) (Saba, 2007; Cataldo et al., 2009). We have recently demonstrated that this epi-drug can demethylate the genome in adult schistosomes and can significantly inhibit the production and maturation of *in vitro* laid eggs (Geyer et al., 2011). In this study, we used RNA-Seq as a hypothesis generating tool to identify transcripts/pathways/features differentially modulated in adult *S. mansoni* females exposed to this ribonucleoside analogue (Fig. 1). This is because it is known that 5-AzaC can also affect other cellular processes, in addition to or because of DNA hypomethylation (Flotho et al., 2009; Hollenbach et al., 2010).

Similar to our previous studies (Geyer et al., 2011), we used a high concentration (491 μM) of 5-AzaC to maximally elicit the egg laying defect in *in vitro* cultivated adult schistosome pairs (Fig. 1A). Here, a 70% reduction in egg output was observed in schistosome pairs (n = 35 pairs) treated with 5-AzaC for 48 h when compared to controls. Also consistent with our previous viability quantification (Geyer et al., 2011), this 5-AzaC mediated anti-fecundity effect was not due to parasite death as both drug treated and control worm pairs remained equally active throughout 7 days of culture, retained gut peristalsis (as indicated by haemozoin movement) and maintained adherence to the tissue culture wells (Fig. 1B and the video in Supplementary Fig. 1).

Supplementary video related to this article can be found at https://doi.org/10.1016/j.ijpddr.2018.03.006

The following is/are the supplementary data related to this article:

Supp Figure1 Supp Figure1

It is currently unclear why 5-AzaC (491 μM) remains nontoxic to adult schistosomes when dose-dependent cytotoxicity has been observed in mammalian cell culture systems (e.g. (Hollenbach et al., 2010)). However, the protozoan parasite *Entamoeba histolytica*, another DNA methyltransferase 2 (DNMT2) only organism (Fisher et al., 2004), also is not affected by high concentrations of 5-AzaC (23 μM) during extended co-cultures (up to 7 days) (Ali et al., 2007). Perhaps due to nucleoside auxotrophy (Levy and Read, 1975a; b), schistosomes (and other parasites like *E. histolytica* (McGugan et al., 2007)) carefully regulate the uptake and transport of these types of (modified) biomolecules from external environments, which limits their toxic accumulation. Alternatively, high concentrations of 5-AzaC are required to affect multi-cellular invertebrates during *in vitro* culture (Vandegehuchte et al., 2010; Geyer et al., 2017), which clearly is not necessary for a mammalian cell monolayer. Nevertheless, the co-cultivation conditions (5-AzaC concentration and duration) established here enabled the detailed analysis of transcriptomes derived from viable drug treated (n = 3 replicates) and control (n = 3 replicates) female schistosomes.

Assessment of biological reproducibility between RNA-Seq replicates demonstrated a strong relationship. Here, performing either Euclidean distance evaluation (Fig. 1C) or principal component analysis (PCA) (Fig. 1D) of differential expression values, clear clustering amongst the replicates was observed. Indeed, PCA assessment showed that almost all variation in experimental outputs (94%) could be explained by 5-AzaC treatment (i.e. biological variability was minimal amongst replicates). Amongst the 4115 differentially expressed transcripts (mapping to 4036 unique Smps) identified in the study, a total of 2109 transcripts (corresponding to 2079 Smps) were significantly down-regulated by 5-AzaC whereas 2006 transcripts (corresponding to 1978 Smps) were significantly up-regulated in adult females (Supplementary Fig. 2). These DEGs were subsequently subjected to functional enrichment and pathway information analyses, which involved classification of Smps using descriptors obtained from Gene Ontology (GO), BioCyc and KEGG.

**Fig. 2:**
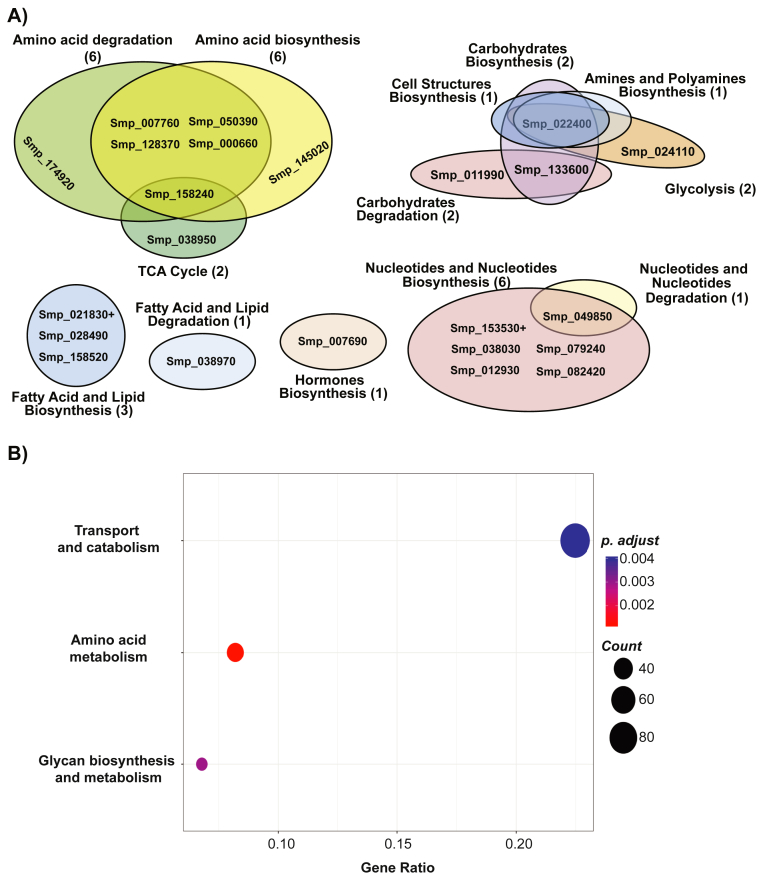
5-**AzaC affects transcripts involved in metabolic processes. A) Venn diagrams of differentially expressed enzymes participating in metabolic pathways were produced using data from BioCyc** (Caspi et al., 2016) **and the Neo4j graph database**. Smps found more abundantly expressed in 5-AzaC treated females are marked with a ‘+’. **B) Scatter plot demonstrating significantly over-represented (*p*** < **0.05) KEGG functional hierarchies by differentially abundant KEGG orthologs (KOs) between 5-AzaC-treated and control females for each dataset.** Circle size represents KO counts within that pathway while red colouration represents lower *p* values and, therefore, higher significance. Gene ratio on the x-axis represents the proportion of genes represented in the pathway to the total number of DEGs. (For interpretation of the references to colour in this figure legend, the reader is referred to the Web version of this article.)

In female schistosomes, GO analysis (using g:Profiler (Reimand et al., 2016)) revealed eleven Biological Process (BP) categories significantly down-regulated by 5-AzaC treatment in comparison to seven BP categories significantly up-regulated (Table 1; Supplementary Fig. 3 also contains details related to Molecular Function and Cellular Component GO categories). Amongst the Smps contained within the BP categories down-regulated by 5-AzaC, many were associated with metabolism of carbohydrates, purines, pyrimidines, ATP and precursors. Conversely, 5-AzaC led to the up-regulation of Smps associated with RNA metabolism and methylation (amongst others). While difficult to directly compare to previous studies examining the effect of 5-AzaC on other eukaryote cell transcriptomes (Ali et al., 2007; Hollenbach et al., 2010; Qiu et al., 2010), our results suggest that 5-AzaC dysregulates a wide-range of metabolic pathways in female schistosomes. To explore this observation further, the statistically significant DEGs were next analysed for metabolic pathway enrichment using BioCyc (Caspi et al., 2016) and KEGG (Kanehisa, 2016) (Fig. 2).

**Table 1:** Gene Ontology (GO) analysis of differentially expressed genes (DEGs).

**Down-regulated in 5-AzaC**		
**Biological Process**	**No. of genes associated to functional term**	**p-value**
nicotinamide nucleotide metabolic process	18	5.39E-3
ATP metabolic process	30	2.20E-5
carbohydrate metabolic process	47	2.53E-4
carbohydrate derivative metabolic process	94	2.06E-6
oxidation-reduction process	77	2.01E-5
pyridine nucleotide metabolic process	18	5.39E-3
pyruvate metabolic process	13	8.11E-3
generation of precursor metabolites and energy	34	6.52E-3
ion transport	92	2.82E-3
transmembrane transport	91	9.63E-4
purine ribonucleoside monophosphate metabolic process	33	1.11E-5

**Up-regulated in 5-AzaC**		
**Biological Process**	**No. of genes associated to functional term**	**p-value**
regulation of gene expression	153	8.65E-4
RNA metabolic process	288	1.68E-14
peptidyl-lysine modification	21	1.83E-2
cell projection assembly	28	3.99E-4
cellular component organization or biogenesis	249	1.72E-6
methylation	28	2.23E-3
mRNA export from nucleus	14	4.57E-2

Gene ontology analysis (Biological Process sub ontology) of DEGs between 5-AzaC-treated and control females using the g:GOSt tool in gProfiler and moderate hierarchical filtering (Reimand et al., 2016). The number of genes associated to each functional term as well as the enrichment *p-value* for each functional category is shown. Enriched Cellular Component and Molecular Function gene ontology categories can also be found in Supplementary Fig. 4.

Consistent with the GO analysis, of the 1074 enzyme reactions included in the BioCyc metabolomics model for *S. mansoni*, 23 Smps (coding for enzymes and participating in 34 reaction pathways) had a log2 fold-change > ±1 and were associated with macromolecule metabolism (amino acids, carbohydrates, fatty acids, lipids, nucleotides/nucleosides and hormones). Interestingly, most (21/23; 91%) of these enzymes were down-regulated by 5-AzaC treatment (Fig. 2A and Supplementary Fig. 4). As well as presenting a vital energy source, these enzymatically produced biomolecules provide important metabolic intermediates for biosynthesis in the parasite and their crucial function in female fecundity has been highlighted in several previous studies (e.g. (Bueding, 1970; Meyer et al., 1970; Schiller et al., 1975; Pearce and Huang, 2015)). Amongst the BioCyc pathways identified, amino acid metabolism contained the greatest number of entries (6 Smps). Interestingly, KEGG analysis also identified amino acid metabolism as the most statistically significant category affected by 5-AzaC in adult females (Fig. 2B and Supplementary Fig. 5).

**Fig. 3:**
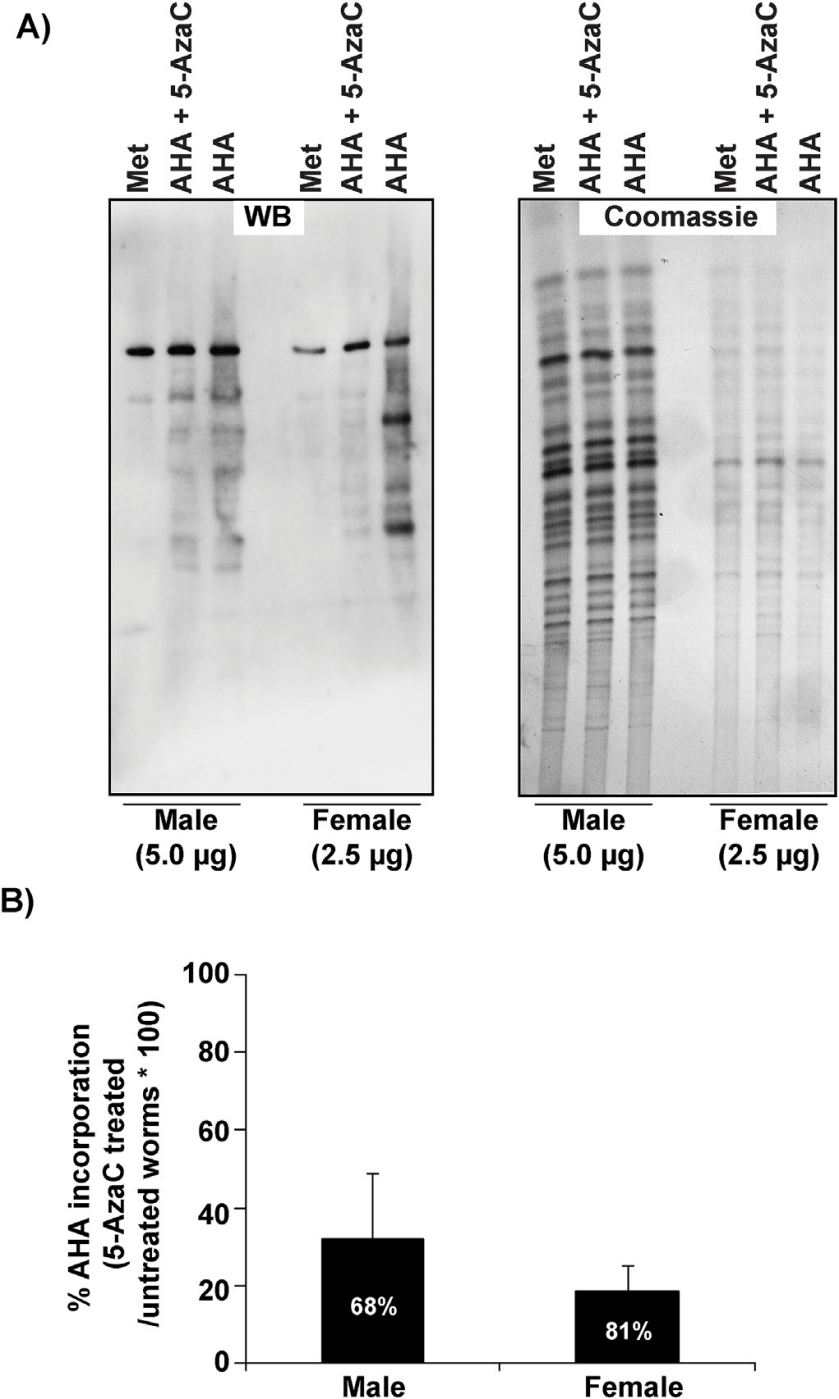
**Protein synthesis in adult male and female schistosomes is inhibited by 5-AzaC. A) 1D gel analysis of nascent protein synthesis in 5-AzaC treated worms.** Male and female worms were incubated with 200 μM AHA in the presence or absence of 491 μM 5-AzaC for 48 h to measure its effect on nascent protein synthesis. Protein extracts were prepared and treated with biotin-alkyne; equal quantities (male samples 5 μg, female samples 2.5 μg) were analysed by SDS-PAGE (4–12% gradient gel) before coomassie blue staining (right), or blotting and probing with streptavidin-HRP (left). Worms were also incubated in 200 μM methionine (Met) and subsequently processed and analysed the same way as a measure of non-specific labelling by streptavidin-HRP. **B) Quantitative analysis of nascent protein synthesis in adult worms incubated with 491 μM 5-AzaC**. ImageJ was used to measure the total pixel intensities of three separate western blots to estimate nascent protein synthesis inhibition mediated by 491 μM 5-AzaC. Values represent percentage of AHA incorporation in 5-AzaC treated worms compared to untreated (AHA only) controls. Error bars represent the standard error of the normalised % incorporation AHA means for each gender.

Hollenbach et al. have previously reported a 5-AzaC-mediated inhibition on nascent protein synthesis in both acute myeloid leukemia KG-1a and THP-1 human cell lines (Hollenbach et al., 2010). Our transcriptome results suggested that this ribonucleoside analogue could be exerting a similar activity in female worms. Therefore, we next investigated whether 5-AzaC mediated dysregulation in transcripts associated with amino acid metabolism correlated to an impact on *de novo* protein synthesis in adult worms (Fig. 3). As previously shown for *Schistosoma japonicum* (Kawanaka and Sugiyama, 1992), the rate of amino acid incorporation in adult *S. mansoni* females was much higher than males in our experiments (compare the WB signal in 5  μg male vs 2.5 μg female samples; Fig. 3A). This gender difference in nascent protein synthesis is due to the increased metabolic demands required by female schistosomes (regardless of species) for egg production (Lawrence, 1973). Despite these baseline differences in amino acid incorporation, we found that 5-AzaC dramatically inhibited this process in both sexes during a 24 h period. Specifically, 5-AzaC led to a 68% and 81% inhibition in *de novo* protein synthesis in male and female schistosomes respectively (Fig. 3B). This inhibitory effect is likely caused by the well-recognised ability of 5-AzaC to incorporate into newly synthesised RNAs (rRNAs, tRNAs, mRNAs and miRNAs), which alters the molecular functions of these ribonucleic acids and negatively affects protein synthesis (Reichman and Penman, 1973; Cihak, 1974; Cihak et al., 1974; Lu and Randerath, 1980). Under these conditions of functional RNA pool depletion, schistosome protein synthesis and egg production is unsustainable.

**Fig. 4:**
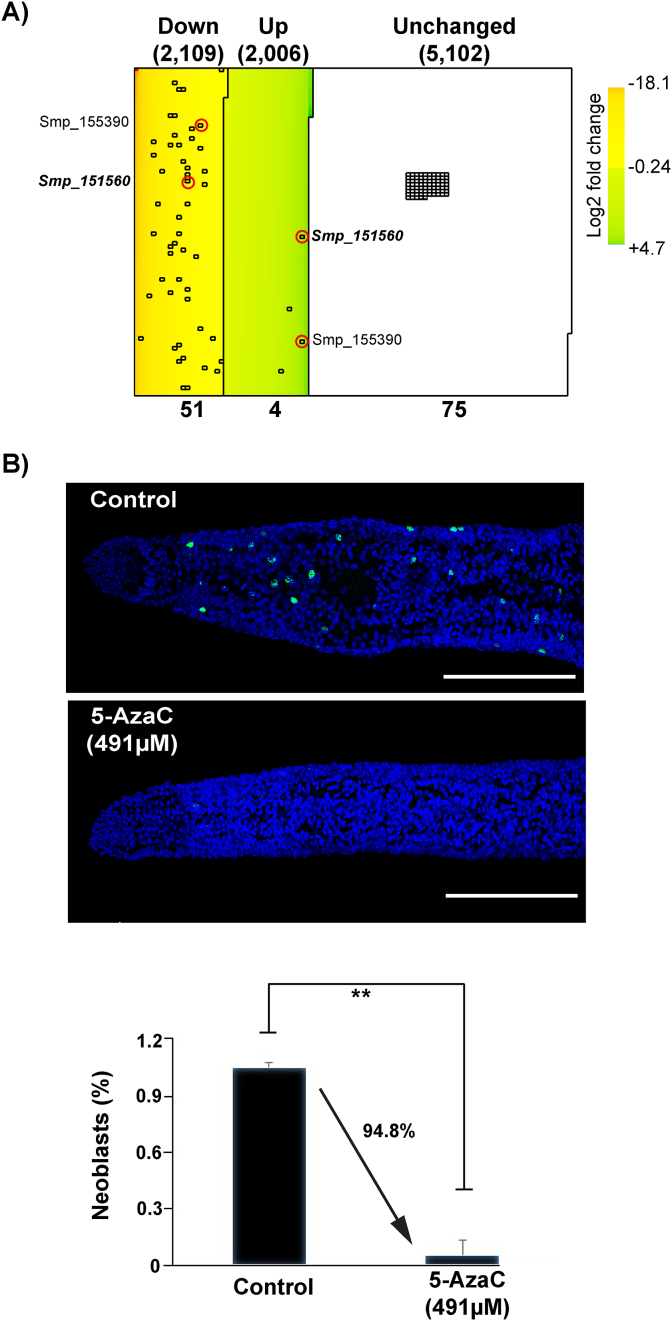
**Stem cell-associated genes affected by 5-AzaC. A) Heatmap of significantly differentially expressed genes, scaled to proportion with normally expressed genes**. First set (Down) of genes (total of 2109) represents transcripts that are downregulated in drug-treated vs. control samples, whereas the second set (Up, total of 2006) are transcripts that demonstrate an upregulation in response to drug treatment. The unchanged category (5,102) represents transcripts which are not significantly differently affected by the drug (adjusted *p*-value < 0.05). Solid squares represent the 128 proliferating cells-enriched transcripts (Collins et al., 2013). Colour reflects expression levels ranging from strongly downregulated genes (dark red square – min. value of −18.1) to strongly upregulated genes (dark green square – max. value of +4.7). Transcripts are ordered based on increasing values of their log_2_ fold change from top-left to bottom-right and each column contains 100 transcripts. Red circles outline two alternatively spliced products, corresponding to the same Smp, found in both downregulated AND upregulated categories. **B) 5-AzaC treatment significantly affects neoblast numbers and proliferation.** Representative anterior ends of female schistosomes treated with 491 μM of 5-AzaC (n = 10) or media controls (n = 6) after a total culture period of 3 days. Bar chart illustrates the percentage of neoblasts present in control (media only) versus 5-AzaC-treated females. Error bars represent standard error of the mean and a two-tailed *t-*test (** corresponds to *p* < 0.001*)* was subsequently performed to explore a statistical difference between the cultures. DAPI = blue; Green = EdU^+^ cells. Size bar represents 100 μm. (For interpretation of the references to colour in this figure legend, the reader is referred to the Web version of this article.)

In addition to stable RNA and protein pools, the critical role of both germinal- and somatic-stem cells in schistosome development as well as egg production has been recently highlighted (Wendt and Collins, 2016). Therefore, the egg-laying deficiency observed during 5-AzaC/worm pair co-cultures (Fig. 1A) could also be caused by this drug modulating (via transcription/translation impairment) stem cell function in adult female schistosomes. Evidence to support this contention originates in studies demonstrating the role of 5-AzaC in promoting stem cell differentiation (Taylor and Jones, 1979; Chen et al., 2015), although this is not always observed and remains a highly-debated subject (Martin-Rendon et al., 2008). Nevertheless, as our previous studies clearly illustrated that, in addition to egg production inhibition, ovaries (containing germinal stem cells) were also negatively affected by 5-AzaC (Geyer et al., 2011), we next investigated whether this epi-drug modulated the differential expression of 128 transcripts enriched in proliferating stem cells (Collins et al., 2013) (Fig. 4; Supplementary Fig. 6). Here, 51 of these stem cell associated transcripts were down-regulated in response to 5-AzaC treatment, compared to 4 that were up-regulated; the expression of the remaining 75 transcripts were unchanged between treatment conditions (Fig. 4A). While it is possible that some of these results (i.e. the unchanged transcripts) can be explained by the gender being examined (here, female worms, whereas Collins et al. examined male worms (Collins et al., 2013)), our data clearly indicated that 5-AzaC negatively impacts female stem cell pools. EdU labelling (24 h pulse) of females treated with 5-AzaC for three days confirmed this observation and revealed the presence of fewer proliferating cells (EdU^+^ nuclei) when compared to controls (Fig. 4B). When quantified at the anterior ends of females, 5-AzaC led to a 95% reduction in the number of proliferating stem cells (Fig. 4C). Further analyses of spatially-distinct stem cell populations (germinal vs somatic) may uncover subtle differences in 5-AzaC-mediated proliferation modulation in female (as well as male) schistosomes. However, this discriminatory analysis was beyond the scope of the current study. Nevertheless, a highly-affected stem cell pool, likely due to dis-regulated transcription and translation, represents another contributing factor associated with the egg-laying defect observed in 5-AzaC-treated schistosomes.

**Fig. 5:**
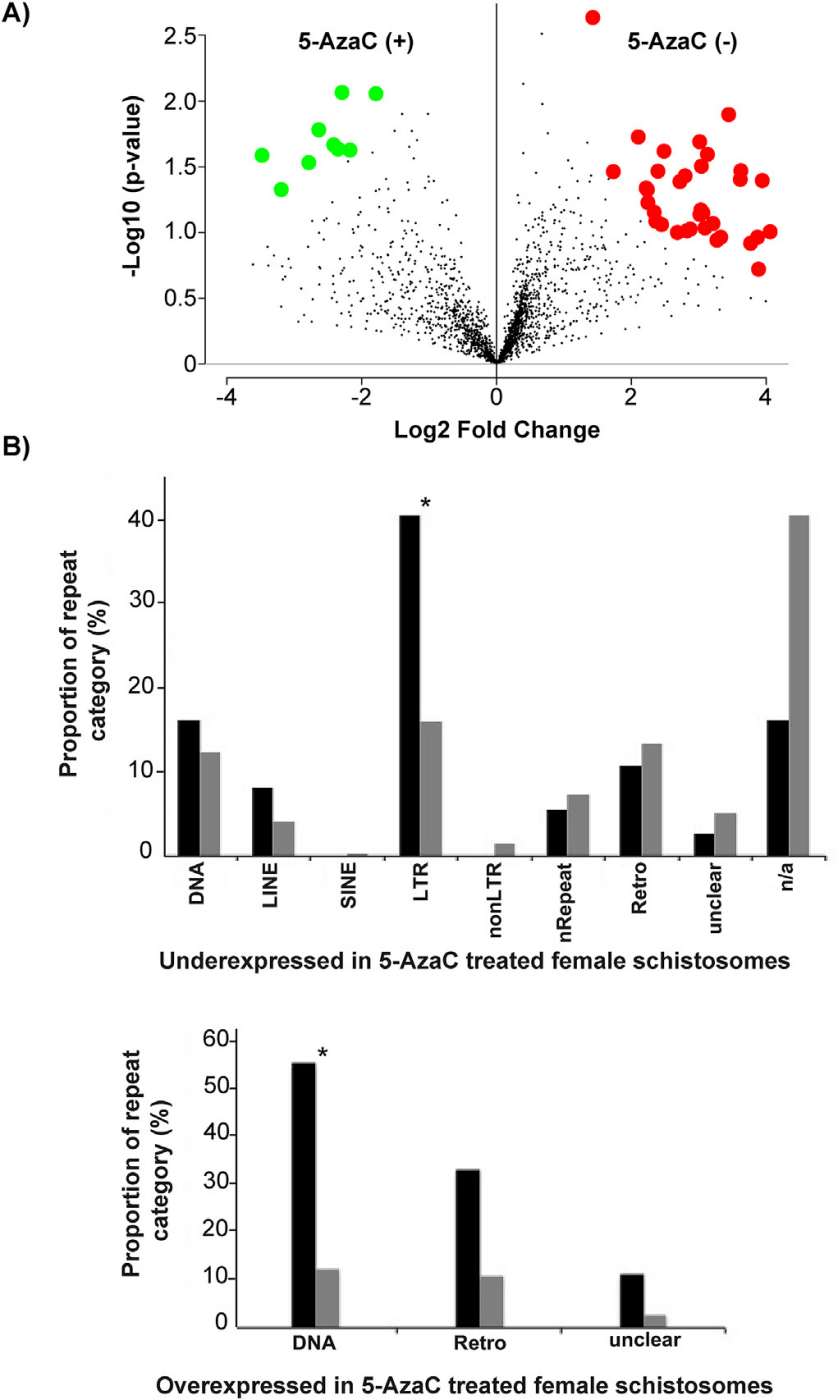
**Analysis of repetitive elements affected by 5-AzaC. A) Volcano plot illustrating differential expression of 2088 repeat subfamilies with counts in both control and 5-AzaC data sets.** Repeat subfamilies with a significant permutation *p*-value (*p* < 0.05) are highlighted. Overexpressed repeat subfamilies (9) in the 5-AzaC samples are indicated by green dots, whereas downregulated repeat subfamilies (37) are marked in red. **B) The demethylating agent significantly affects specific repeat categories.** Relative abundance of the repeat categories (proportion %) which are underexpressed and overexpressed in the presence of 5-AzaC are represented. Black bars indicate the proportion (%) of repeat categories, whereas grey bars demonstrate the proportion of the represented class across the entire genome. Statistical significance was assessed using a two-proportions z test (*p* = 0.011 for DNA and *p* = 0.003 for LTR) and Holm-Bonferroni for multiple comparison corrections. Nomenclature for repeat classifications is identical to that described by Lepesant et al. (Lepesant et al., 2012) except for nRepeat, which defines tandom repeat classes (e.g. ATATn or TGCn). (For interpretation of the references to colour in this figure legend, the reader is referred to the Web version of this article.)

In addition to 5-AzaC‘s recognised role in modulating the transcription of protein coding genes (e.g.(Hollenbach et al., 2010), this study), this epi-drug has also been shown to affect the expression of endogenous retroviruses (ERVs) and repetitive elements (REs) in ovarian cell line (TykNu and Hey) or MDS primary cell (CD34^+^ progenitors) cultures (Chiappinelli et al., 2015; Tobiasson et al., 2017). Therefore, dysregulated RE expression in 5-AzaC-treated schistosomes could also be responsible for the egg laying phenotype observed here as well as in previous studies (Geyer et al., 2011). Examination of the transcriptome dataset revealed the differential expression of 2088 REs between control and drug treated female samples (Fig. 5 and Supplementary Fig. 7). Amongst these, only 9 REs (represented as solid green circles) were significantly enriched in the 5-AzaC-treated female samples compared to 37 REs (represented as solid red circles) significantly enriched in the control samples (Fig. 5A). As a large proportion of the *S. mansoni* genome is occupied by repeats (Berriman et al., 2009; Lepesant et al., 2012), we identified the significantly enriched RE categories by comparing their occurrence in our transcriptome dataset to their occurrence in the genome. By doing so, only two types of RE in 5-AzaC-treated female schistosomes were identified as having statistically significant changes in transcription: long terminal repeats (LTR) are under-expressed (*p* = 0.003) and DNA transposons are over-expressed (*p* = 0.011) (Fig. 5B). The significance of these findings is unclear, but as more than 33% of the schistosome RE repertoire is transcriptionally active (Lepesant et al., 2012), our results indicate that normal schistosome oviposition is somehow associated with controlled RE expression. How 5-AzaC modulates schistosome RE expression is also currently unknown, but may be related to DNA demethylation (i.e. a cercarial RE was previously found to be methylated (Geyer et al., 2011)) or some other molecular process that disrupts ribonucleic acid pools (Li et al., 1970), histone modifications (So et al., 2011) and protein metabolism (as indicated herein). Nevertheless, transcriptional control of these RE genomic features is likely critical for maintaining normal developmental features (including egg-laying) throughout the schistosome lifecycle.

In this study, we extend our understanding of the anti-fecundity activity of 5-AzaC on *S. mansoni.* By disrupting both transcription and translation, this ribonucleoside analogue affects the downstream processes of stem cell biology, RE maintenance and schistosome egg production in a broad-acting, pleiotropic manner. In addition to DNA methylation (Geyer et al., 2011), these core molecular activities are indispensable for maintaining oviposition in mature schistosome pairs. Future studies aiming to target any one of these essential activities (summarised in Supplementary Fig. 8) could direct a more specific approach in inhibiting schistosome egg production and, thus, offer a new strategy for controlling schistosomiasis immunopathology and transmission.

## Conflicts of interest

The authors certify that they have NO affiliations with or involvement in any organization or entity with any financial interest (such as honoraria; educational grants; participation in speakers’ bureaus; membership, employment, consultancies, stock ownership, or other equity interest; and expert testimony or patent-licensing arrangements), or non-financial interest (such as personal or professional relationships, affiliations, knowledge or beliefs) in the subject matter or materials discussed in this manuscript.

## Appendix A Supplementary data

Data Profile

Supp Figure2 Supp Figure2

Supp Figure3 Supp Figure3

Supp Figure4 Supp Figure4

Supp Figure5 Supp Figure5

Supp Figure6 Supp Figure6

Supp Figure7 Supp Figure7

Supp Figure8 Supp Figure8

Supp figure legends Supp figure legends

## References

[bib1] Ali I.K., Ehrenkaufer G.M., Hackney J.A., Singh U. (2007). Growth of the protozoan parasite *Entamoeba histolytica* in 5-azacytidine has limited effects on parasite gene expression. BMC Genom..

[bib2] Anders S., Huber W. (2010). Differential expression analysis for sequence count data. Genome Biol..

[bib3] Andrews S. (2010). FASTQC. http://www.bioinformatics.babraham.ac.uk/projects/fastqc/.

[bib4] Berriman M., Haas B.J., LoVerde P.T., Wilson R.A., Dillon G.P., Cerqueira G.C., Mashiyama S.T., Al-Lazikani B., Andrade L.F., Ashton P.D., Aslett M.A., Bartholomeu D.C., Blandin G., Caffrey C.R., Coghlan A., Coulson R., Day T.A., Delcher A., DeMarco R., Djikeng A., Eyre T., Gamble J.A., Ghedin E., Gu Y., Hertz-Fowler C., Hirai H., Hirai Y., Houston R., Ivens A., Johnston D.A., Lacerda D., Macedo C.D., McVeigh P., Ning Z., Oliveira G., Overington J.P., Parkhill J., Pertea M., Pierce R.J., Protasio A.V., Quail M.A., Rajandream M.-A., Rogers J., Sajid M., Salzberg S.L., Stanke M., Tivey A.R., White O., Williams D.L., Wortman J., Wu W., Zamanian M., Zerlotini A., Fraser-Liggett C.M., Barrell B.G., El-Sayed N.M. (2009). The genome of the blood fluke *Schistosoma mansoni*. Nature.

[bib5] Blohm A.S., Mader P., Quack T., Lu Z., Hahnel S., Schlitzer M., Grevelding C.G. (2016). Derivatives of biarylalkyl carboxylic acid induce pleiotropic phenotypes in adult *Schistosoma mansoni in vitro*. Parasitol. Res..

[bib6] Bueding E. (1970). Physiology and biochemistry of schistosomes. J. Parasitol..

[bib7] Carneiro V.C., de Abreu da Silva I.C., Torres E.J., Caby S., Lancelot J., Vanderstraete M., Furdas S.D., Jung M., Pierce R.J., Fantappie M.R. (2014). Epigenetic changes modulate schistosome egg formation and are a novel target for reducing transmission of schistosomiasis. PLoS Pathog..

[bib8] Caspi R., Billington R., Ferrer L., Foerster H., Fulcher C.A., Keseler I.M., Kothari A., Krummenacker M., Latendresse M., Mueller L.A., Ong Q., Paley S., Subhraveti P., Weaver D.S., Karp P.D. (2016). The MetaCyc database of metabolic pathways and enzymes and the BioCyc collection of pathway/genome databases. Nucleic Acids Res..

[bib9] Cass C.L., Johnson J.R., Califf L.L., Xu T., Hernandez H.J., Stadecker M.J., Yates J.R., Williams D.L. (2007). Proteomic analysis of *Schistosoma mansoni* egg secretions. Mol. Biochem. Parasitol..

[bib10] Cataldo V.D., Cortes J., Quintas-Cardama A. (2009). Azacitidine for the treatment of myelodysplastic syndrome. Expert Rev. Anticancer Ther..

[bib11] Chen G., Yue A., Ruan Z., Yin Y., Wang R., Ren Y., Zhu L. (2015). Potential of 5-azacytidine induction decidual stromal cells from maternal human term placenta towards cardiomyocyte-like cells in serum-free medium. Cell Tissue Bank..

[bib12] Chiappinelli K.B., Strissel P.L., Desrichard A., Li H., Henke C., Akman B., Hein A., Rote N.S., Cope L.M., Snyder A., Makarov V., Budhu S., Slamon D.J., Wolchok J.D., Pardoll D.M., Beckmann M.W., Zahnow C.A., Merghoub T., Chan T.A., Baylin S.B., Strick R. (2015). Inhibiting DNA methylation causes an interferon response in cancer via dsRNA including endogenous retroviruses. Cell.

[bib13] Cihak A. (1974). Biological effects of 5-azacytidine in eukaryotes. Oncology.

[bib14] Cihak A., Weiss J.W., Pitot H.C. (1974). Characterization of polyribosomes and maturation of ribosomal RNA in hepatoma cells treated with 5-azacytidine. Canc. Res..

[bib15] Collins J.J., Wang B., Lambrus B.G., Tharp M.E., Iyer H., Newmark P.A. (2013). Adult somatic stem cells in the human parasite *Schistosoma mansoni*. Nature.

[bib16] Cosseau C., Wolkenhauer O., Padalino G., Geyer K.K., Hoffmann K.F., Grunau C. (2016). (Epi)genetic inheritance in *Schistosoma mansoni*: a systems approach. Trends Parasitol..

[bib17] Criscione S.W., Zhang Y., Thompson W., Sedivy J.M., Neretti N. (2014). Transcriptional landscape of repetitive elements in normal and cancer human cells. BMC Genom..

[bib18] Edwards J., Brown M., Peak E., Bartholomew B., Nash R.J., Hoffmann K.F. (2015). The diterpenoid 7-keto-sempervirol, derived from lycium chinense, displays anthelmintic activity against both *Schistosoma mansoni* and *Fasciola hepatica*. PLoS Neglected Trop. Dis..

[bib19] Ferreira M.S., de Oliveira D.N., de Oliveira R.N., Allegretti S.M., Catharino R.R. (2014). Screening the life cycle of *Schistosoma mansoni* using high-resolution mass spectrometry. Anal. Chim. Acta.

[bib20] Fisher O., Siman-Tov R., Ankri S. (2004). Characterization of cytosine methylated regions and 5-cytosine DNA methyltransferase (Ehmeth) in the protozoan parasite *Entamoeba histolytica*. Nucleic Acids Res..

[bib21] Fitzpatrick J.M., Hirai Y., Hirai H., Hoffmann K.F. (2007). Schistosome egg production is dependent upon the activities of two developmentally regulated tyrosinases. Faseb. J..

[bib22] Fitzpatrick J.M., Hoffmann K.F. (2006). Dioecious *Schistosoma mansoni* express divergent gene repertoires regulated by pairing. Int. J. Parasitol..

[bib23] Fitzpatrick J.M., Peak E., Perally S., Chalmers I.W., Barrett J., Yoshino T.P., Ivens A.C., Hoffmann K.F. (2009). Anti-schistosomal intervention targets identified by lifecycle transcriptomic analyses. PLoS Neglected Trop. Dis..

[bib24] Flotho C., Claus R., Batz C., Schneider M., Sandrock I., Ihde S., Plass C., Niemeyer C.M., Lubbert M. (2009). The DNA methyltransferase inhibitors azacitidine, decitabine and zebularine exert differential effects on cancer gene expression in acute myeloid leukemia cells. Leukemia.

[bib25] Geyer K.K., Hoffmann K.F. (2012). Epigenetics: a key regulator of platyhelminth developmental biology?. Int. J. Parasitol..

[bib26] Geyer K.K., Niazi U.H., Duval D., Cosseau C., Tomlinson C., Chalmers I.W., Swain M.T., Cutress D.J., Bickham-Wright U., Munshi S.E., Grunau C., Yoshino T.P., Hoffmann K.F. (2017). The *Biomphalaria glabrata* DNA methylation machinery displays spatial tissue expression, is differentially active in distinct snail populations and is modulated by interactions with Schistosoma mansoni. PLoS Neglected Trop. Dis..

[bib27] Geyer K.K., Rodriguez Lopez C.M., Chalmers I.W., Munshi S.E., Truscott M., Heald J., Wilkinson M.J., Hoffmann K.F. (2011). Cytosine methylation regulates oviposition in the pathogenic blood fluke *Schistosoma mansoni*. Nat. Commun..

[bib28] Hoffmann K.F., Strand M. (1997). Molecular characterization of a 20.8-kDa *Schistosoma mansoni* antigen. Sequence similarity to tegumental associated antigens and dynein light chains. J. Biol. Chem..

[bib29] Hoffmann K.F., Wynn T.A., Dunne D.W. (2002). Cytokine-mediated host responses during schistosome infections; walking the fine line between immunological control and immunopathology. Adv. Parasitol..

[bib30] Hokke C.H., Fitzpatrick J.M., Hoffmann K.F. (2007). Integrating transcriptome, proteome and glycome analyses of *Schistosoma* biology. Trends Parasitol..

[bib31] Hollenbach P.W., Nguyen A.N., Brady H., Williams M., Ning Y., Richard N., Krushel L., Aukerman S.L., Heise C., MacBeth K.J. (2010). A comparison of azacitidine and decitabine activities in acute myeloid leukemia cell lines. PLoS One.

[bib32] Howe K.L., Bolt B.J., Cain S., Chan J., Chen W.J., Davis P., Done J., Down T., Gao S., Grove C., Harris T.W., Kishore R., Lee R., Lomax J., Li Y., Muller H.M., Nakamura C., Nuin P., Paulini M., Raciti D., Schindelman G., Stanley E., Tuli M.A., Van Auken K., Wang D., Wang X., Williams G., Wright A., Yook K., Berriman M., Kersey P., Schedl T., Stein L., Sternberg P.W. (2016). WormBase 2016: expanding to enable helminth genomic research. Nucleic Acids Res..

[bib33] Howe K.L., Bolt B.J., Shafie M., Kersey P., Berriman M. (2017). WormBase ParaSite - a comprehensive resource for helminth genomics. Mol. Biochem. Parasitol..

[bib34] Kanehisa M. (2016). KEGG bioinformatics resource for plant genomics and metabolomics. Meth. Mol. Biol..

[bib35] Kawanaka M., Sugiyama H. (1992). Incorporation of radiolabelled amino acids by adult *Schistosoma japonicum*: further characterization of a putative eggshell precursor protein. Int. J. Parasitol..

[bib36] Krzywinski M., Schein J., Birol I., Connors J., Gascoyne R., Horsman D., Jones S.J., Marra M.A. (2009). Circos: an information aesthetic for comparative genomics. Genome Res..

[bib37] Lawrence J.D. (1973). The ingestion of red blood cells by *Schistosoma mansoni*. J. Parasitol..

[bib38] Lepesant J.M., Roquis D., Emans R., Cosseau C., Arancibia N., Mitta G., Grunau C. (2012). Combination of de novo assembly of massive sequencing reads with classical repeat prediction improves identification of repetitive sequences in *Schistosoma mansoni*. Exp. Parasitol..

[bib39] Levy M.G., Read C.P. (1975). Purine and pyrimidine transport in *Schistosoma mansoni*. J. Parasitol..

[bib40] Levy M.G., Read C.P. (1975). Relation of tegumentary phosphohydrolase to purine and pyrimidine transport in *Schistosoma mansoni*. J. Parasitol..

[bib41] Li L.H., Olin E.J., Buskirk H.H., Reineke L.M. (1970). Cytotoxicity and mode of action of 5-azacytidine on L1210 leukemia. Canc. Res..

[bib42] Lu L.J., Randerath K. (1980). Mechanism of 5-azacytidine-induced transfer RNA cytosine-5-methyltransferase deficiency. Canc. Res..

[bib43] Lu Z., Sessler F., Holroyd N., Hahnel S., Quack T., Berriman M., Grevelding C.G. (2016). Schistosome sex matters: a deep view into gonad-specific and pairing-dependent transcriptomes reveals a complex gender interplay. Sci. Rep..

[bib44] Lu Z., Sessler F., Holroyd N., Hahnel S., Quack T., Berriman M., Grevelding C.G. (2017). A gene expression atlas of adult *Schistosoma mansoni* and their gonads. Sci Data.

[bib45] Majek P., Riedelova-Reicheltova Z., Suttnar J., Dyr J.E. (2013). Staining of proteins for 2D SDS-PAGE using Coomassie Blue–speed versus sensitivity?. Electrophoresis.

[bib46] Marcellino C., Gut J., Lim K.C., Singh R., McKerrow J., Sakanari J. (2012). WormAssay: a novel computer application for whole-plate motion-based screening of macroscopic parasites. PLoS Neglected Trop. Dis..

[bib47] Martin-Rendon E., Sweeney D., Lu F., Girdlestone J., Navarrete C., Watt S.M. (2008). 5-Azacytidine-treated human mesenchymal stem/progenitor cells derived from umbilical cord, cord blood and bone marrow do not generate cardiomyocytes in vitro at high frequencies. Vox Sang..

[bib48] McGugan G.C., Joshi M.B., Dwyer D.M. (2007). Identification and biochemical characterization of unique secretory nucleases of the human enteric pathogen, *Entamoeba histolytica*. J. Biol. Chem..

[bib49] Meyer F., Meyer H., Bueding E. (1970). Lipid metabolism in the parasitic and free-living flatworms, *Schistosoma mansoni* and *Dugesia dorotocephala*. Biochim. Biophys. Acta.

[bib50] Morel M., Vanderstraete M., Hahnel S., Grevelding C.G., Dissous C. (2014). Receptor tyrosine kinases and schistosome reproduction: new targets for chemotherapy. Front. Genet..

[bib51] Pearce E.J., Huang S.C. (2015). The metabolic control of schistosome egg production. Cell Microbiol..

[bib52] Picard M.A., Boissier J., Roquis D., Grunau C., Allienne J.F., Duval D., Toulza E., Arancibia N., Caffrey C.R., Long T., Nidelet S., Rohmer M., Cosseau C. (2016). Sex-biased transcriptome of *Schistosoma mansoni*: host-parasite interaction, genetic determinants and epigenetic regulators are associated with sexual differentiation. PLoS Neglected Trop. Dis..

[bib53] Poirier S., Samami S., Mamarbachi M., Demers A., Chang T.Y., Vance D.E., Hatch G.M., Mayer G. (2014). The epigenetic drug 5-azacytidine interferes with cholesterol and lipid metabolism. J. Biol. Chem..

[bib54] Qiu X., Hother C., Ralfkiaer U.M., Sogaard A., Lu Q., Workman C.T., Liang G., Jones P.A., Gronbaek K. (2010). Equitoxic doses of 5-azacytidine and 5-aza-2'deoxycytidine induce diverse immediate and overlapping heritable changes in the transcriptome. PLoS One.

[bib55] Raddatz G., Guzzardo P.M., Olova N., Fantappie M.R., Rampp M., Schaefer M., Reik W., Hannon G.J., Lyko F. (2013). Dnmt2-dependent methylomes lack defined DNA methylation patterns. Proc. Natl. Acad. Sci. U.S.A..

[bib56] Reichman M., Penman S. (1973). The mechanism of inhibition of protein synthesis by 5-azacytidine in HeLa cells. Biochim. Biophys. Acta.

[bib57] Reimand J., Arak T., Adler P., Kolberg L., Reisberg S., Peterson H., Vilo J. (2016). g:Profiler-a web server for functional interpretation of gene lists (2016 update). Nucleic Acids Res..

[bib58] Saba H.I. (2007). Decitabine in the treatment of myelodysplastic syndromes. Therapeut. Clin. Risk Manag..

[bib59] Schiller E.L., Bueding E., Turner V.M., Fisher J. (1975). Aerobic and anaerobic carbohydrate metabolism and egg production of *Schistosoma mansoni* in vitro. J. Parasitol..

[bib60] Smit C.H., van Diepen A., Nguyen D.L., Wuhrer M., Hoffmann K.F., Deelder A.M., Hokke C.H. (2015). Glycomic analysis of life stages of the human parasite *Schistosoma mansoni* reveals developmental expression profiles of functional and antigenic glycan motifs. Mol. Cell. Proteomics.

[bib61] Smithers S.R., Terry R.J. (1965). The infection of laboratory hosts with cercariae of *Schistosoma mansoni* and the recovery of the adult worms. Parasitology.

[bib62] So A.Y., Jung J.W., Lee S., Kim H.S., Kang K.S. (2011). DNA methyltransferase controls stem cell aging by regulating BMI1 and EZH2 through microRNAs. PLoS One.

[bib63] Sorm F., Piskala A., Cihak A., Vesely J. (1964). 5-Azacytidine, a new, highly effective cancerostatic. Experientia.

[bib64] Sotillo J., Doolan D., Loukas A. (2017). Recent advances in proteomic applications for schistosomiasis research: potential clinical impact. Expert Rev. Proteomics.

[bib65] Standley C.J., Mugisha L., Dobson A.P., Stothard J.R. (2012). Zoonotic schistosomiasis in non-human primates: past, present and future activities at the human-wildlife interface in Africa. J. Helminthol..

[bib66] Stresemann C., Lyko F. (2008). Modes of action of the DNA methyltransferase inhibitors azacytidine and decitabine. Int. J. Canc..

[bib67] Strick R., Strissel P.L., Baylin S.B., Chiappinelli K.B. (2016). Unraveling the molecular pathways of DNA-methylation inhibitors: human endogenous retroviruses induce the innate immune response in tumors. OncoImmunology.

[bib68] Taylor S.M., Jones P.A. (1979). Multiple new phenotypes induced in 10T1/2 and 3T3 cells treated with 5-azacytidine. Cell.

[bib69] Taylor S.M., Jones P.A. (1982). Mechanism of action of eukaryotic DNA methyltransferase. Use of 5-azacytosine-containing DNA. J. Mol. Biol..

[bib70] Tobiasson M., Abdulkadir H., Lennartsson A., Katayama S., Marabita F., De Paepe A., Karimi M., Krjutskov K., Einarsdottir E., Grovdal M., Jansson M., Ben Azenkoud A., Corddedu L., Lehmann S., Ekwall K., Kere J., Hellstrom-Lindberg E., Ungerstedt J. (2017). Comprehensive mapping of the effects of azacitidine on DNA methylation, repressive/permissive histone marks and gene expression in primary cells from patients with MDS and MDS-related disease. Oncotarget.

[bib71] Truscott M., Evans D.A., Gunn M., Hoffmann K.F. (2013). *Schistosoma mansoni* hemozoin modulates alternative activation of macrophages via specific suppression of retnla expression and secretion. Infect. Immun..

[bib72] Vandegehuchte M.B., Lemiere F., Vanhaecke L., Vanden Berghe W., Janssen C.R. (2010). Direct and transgenerational impact on *Daphnia magna* of chemicals with a known effect on DNA methylation. Comp. Biochem. Physiol. C Toxicol. Pharmacol..

[bib73] Warren K.S. (1978). The pathology, pathobiology and pathogenesis of schistosomiasis. Nature.

[bib74] Webber J. (2012). A programmatic introduction to Neo4j. Proceedings of the 3rd annual conference on Systems, programming, and applications: software for humanity.

[bib75] Wendt G.R., Collins J.J. (2016). Schistosomiasis as a disease of stem cells. Curr. Opin. Genet. Dev..

[bib76] Wu X.J., Sabat G., Brown J.F., Zhang M., Taft A., Peterson N., Harms A., Yoshino T.P. (2009). Proteomic analysis of *Schistosoma mansoni* proteins released during in vitro miracidium-to-sporocyst transformation. Mol. Biochem. Parasitol..

[bib77] Xiao Y., Hsiao T.H., Suresh U., Chen H.I., Wu X., Wolf S.E., Chen Y. (2014). A novel significance score for gene selection and ranking. Bioinformatics.

[bib78] Yu G., Wang L.G., Han Y., He Q.Y. (2012). clusterProfiler: an R package for comparing biological themes among gene clusters. OMICS.

