# Corrigendum to ‘the anti-fecundity effect of 5-azacytidine (5-AzaC) on Schistosoma mansoni is linked to dis-regulated transcription, translation and stem cell activities’ [Int. J. Parasitol. Drugs and Drug Resist. 8 (2018) 213–222]

**DOI:** 10.1016/j.ijpddr.2018.07.002

**Published:** 2018-07-17

**Authors:** Kathrin K. Geyer, Sabrina E. Munshi, Martin Vickers, Michael Squance, Toby J. Wilkinson, Daniel Berrar, Cristian Chaparro, Martin T. Swain, Karl F. Hoffmann

**Affiliations:** a Institute of Biological, Environmental and Rural Sciences (IBERS), Edward Llwyd Building, Aberystwyth University, Aberystwyth, SY23 3DA, United Kingdom; b Department of Cell and Developmental Biology, John Innes Centre, Norwich Research Park, Norwich, NR4 7UH, United Kingdom; c The Roslin Institute, The University of Edinburgh, Easter Bush Campus, Midlothian, EH25 9RG, United Kingdom; d Data Science Laboratory, Tokyo Institute of Technology, Tokyo, 152-8550, Japan; e University of Perpignan Via Domitia, 58 Avenue Paul Alduy, Bat R, F-66860, Perpignan Cedex, France

The authors regret that the text describing the filtering of the RNA-Seq data contains an inaccuracy. We would like to correct this information in two places within the manuscript.

In the Materials and Methods section 2.5, 6th sentence, it should read: “Here, 4115 differentially expressed transcripts mapping to 4036 unique Smp gene identifiers were classified.”

In the Materials and methods section 2.6, 10th sentence, it should read: “To illustrate an overview of the genome locations and differential expression levels of the 4057 Smps (4115 transcripts) in the filtered RNA-Seq dataset, a Circos plot (Krzywinski et al., 2009) was generated using the S. mansoni genome v 5.2.”

The authors would like to apologise for any inconvenience caused.

