# Supplementary figures and images for: The anti-fecundity effect of 5-azacytidine (5-AzaC) on Schistosoma mansoni is linked to dis-regulated transcription, translation and stem cell activities
Source: Int J Parasitol Drugs Drug Resist. 2018 Apr 1;8(2):213–22. doi: 10.1016/j.ijpddr.2018.03.006 (PMC6039303; doi:10.1016/j.ijpddr.2018.03.006)

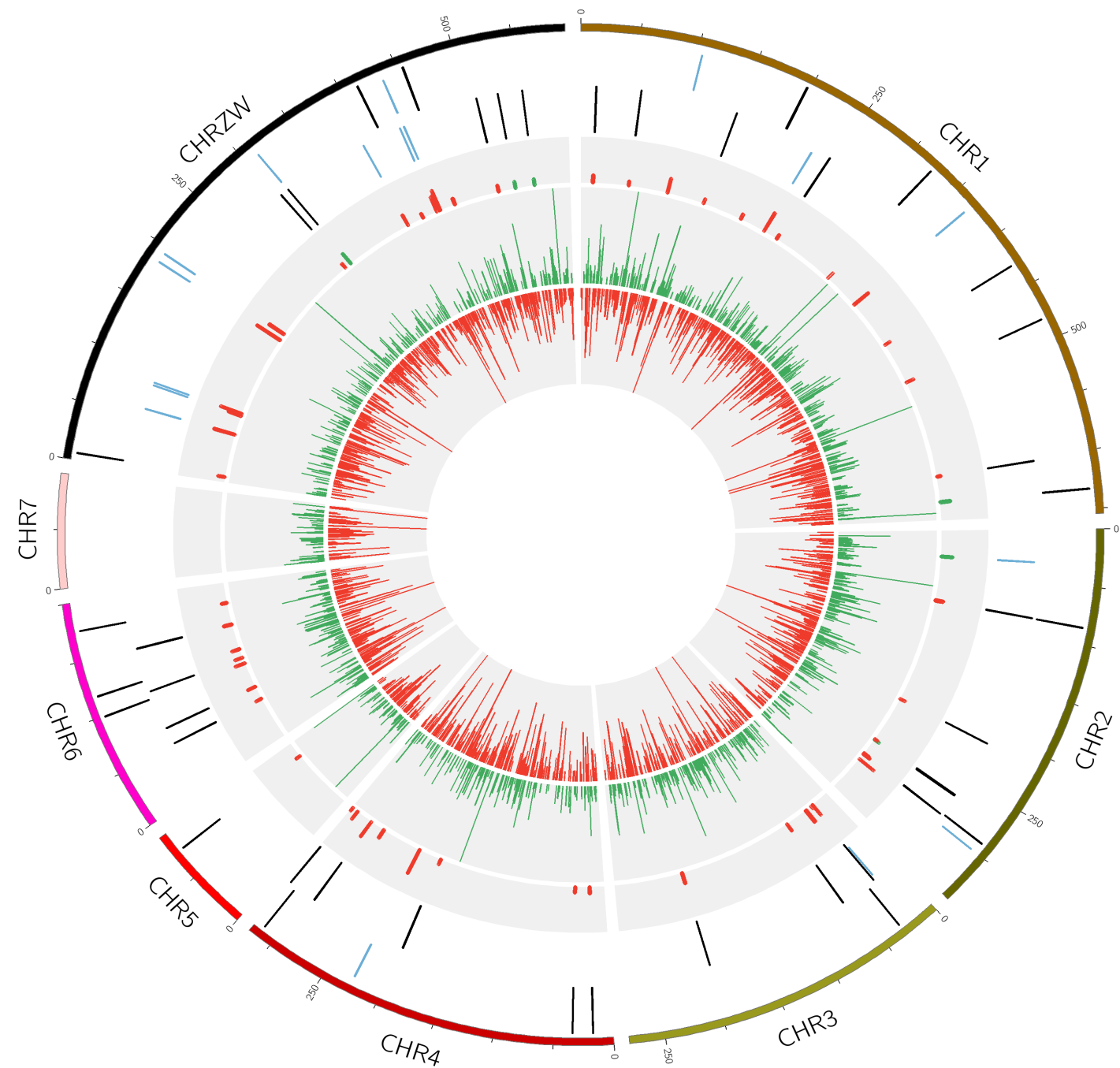

Supplement: Supp Figure8 [file mmc8.pdf]
