# Supplementary material for: The anti-fecundity effect of 5-azacytidine (5-AzaC) on Schistosoma mansoni is linked to dis-regulated transcription, translation and stem cell activities
Source: Int J Parasitol Drugs Drug Resist. 2018 Apr 1;8(2):213–22. doi: 10.1016/j.ijpddr.2018.03.006 (PMC6039303; doi:10.1016/j.ijpddr.2018.03.006)
Supplement: Supp figure legends [file mmc9.docx]

**Supplementary Data**

**Supplementary Figure 1. 5-AzaC does not kill adult schistosome worms.** Five adult worm pairs were cultured either in the presence or absence of 491µM 5-AzaC for up to seven days. Representative videos of 5-AzaC treated- **(A)** and control- **(B)** pairs cultivated for seven days are provided.

**Supplementary Figure 2. Differential expression analysis.** Differentially expressed genes (DEGs) between 5-AzaC-treated and control females were identified using DESeq2 with an FDR of 0.05 (using Benjamini-Hochberg correction) and Smp IDs were subsequently assigned to the transcripts. Smps highlighted in red are represented more than once and likely represent alternatively spliced versions of the gene.

**Supplementary Figure 3. Gene Ontology (GO) analysis of DEGs.** gProfiler output files of GO analysis of DEGs between 5-AzaC-treated and control females using the g:GOSt tool and moderate hierarchical filtering (Reimand et al., 2016). Two separate tabs indicate those GO terms (containing Smps, *S. mansoni* genome v5.2) significantly enriched in either 5-AzaC or control female samples. BP = biological processes, CC = cell component, MF = molecular function.

**Supplementary Figure 4. BioCyc analysis of DEGs.** Those Smps (*S. mansoni* genome v5.2), aligned to metabolic pathway, found more abundantly expressed (log2 fold change > ±1, tab 1; log2 fold change> ±0, tab 2) in either 5-AzaC (positive values) or control (negative values) females by BioCyc analysis (Caspi et al., 2016) are indicated.

**Supplementary Figure 5. Kyoto encyclopedia of genes and genomes (KEGG) pathway analysis of DEGs.** KEGG BRITE (Kanehisa, 2016) functional hierarchical information for each DEG with a mapped SMP ID was extracted. KEGG over-representation analysis (cut off *p*<0.05) was performed on DEGs using the Bioconductor packages clusterProfiler (Yu et al., 2012) in R. *p*-values were corrected for multiple testing using the Benjamini-Hochberg method.

**Supplementary Figure 6. Differential expression of neoblast associated transcripts.** Genes (Smps = 128; *S. mansoni* v5.2) associated with neoblast function were obtained (Collins et al., 2013). The ability of 5-AzaC to affect their transcriptional abundance in adult females was subsequently assessed (compared to controls). n/a = uncharacterised protein. Smps highlighted in yellow represent those where RNA-Seq reads have likely mapped to alternatively spliced products.

**Supplementary Figure 7. Analysis of differentially expressed repetitive elements.** Of the 2,088 differentially expressed repeat subfamilies, 46 contain a significant permutation *p*-value (*p*<0.05; see Materials and Methods) and are represented here. Nomenclature for repeat classification is identical to that described by Lepesant *et al*. (Lepesant et al., 2012).

**Supplementary Figure 8. Circos plot summarising the RNA-Seq dataset.** A Circos plot (Krzywinski et al., 2009) was generated for the entire *S. mansoni* genome (v5.2) spread across seven autosomes and the sex-defining Z/W chimeric allosome. The two outermost rings indicate the genome locations on either DNA strand (+ = inner; - = outer) for the differentially expressed stem cell genes (black) and metabolic network genes (blue). The two innermost rings are the log2 fold-change values for all differentially expressed genes in the genome: in the drug-treated sample the green ring represents up-regulated genes and the red ring down-regulated genes. The middle red and green ring represent the log2 fold-change values for each of the genes in the outermost rings (green for up-regulated, red for down-regulated as before).

**References**

Caspi, R., Billington, R., Ferrer, L., Foerster, H., Fulcher, C.A., Keseler, I.M., Kothari, A., Krummenacker, M., Latendresse, M., Mueller, L.A., Ong, Q., Paley, S., Subhraveti, P., Weaver, D.S., Karp, P.D., 2016. The MetaCyc database of metabolic pathways and enzymes and the BioCyc collection of pathway/genome databases. Nucleic Acids Res 44, D471-480.

Collins, J.J., 3rd, Wang, B., Lambrus, B.G., Tharp, M.E., Iyer, H., Newmark, P.A., 2013. Adult somatic stem cells in the human parasite Schistosoma mansoni. Nature 494, 476-479.

Kanehisa, M., 2016. KEGG Bioinformatics Resource for Plant Genomics and Metabolomics. Methods Mol Biol 1374, 55-70.

Lepesant, J.M., Roquis, D., Emans, R., Cosseau, C., Arancibia, N., Mitta, G., Grunau, C., 2012. Combination of de novo assembly of massive sequencing reads with classical repeat prediction improves identification of repetitive sequences in Schistosoma mansoni. Exp Parasitol 130, 470-474.

Reimand, J., Arak, T., Adler, P., Kolberg, L., Reisberg, S., Peterson, H., Vilo, J., 2016. g:Profiler-a web server for functional interpretation of gene lists (2016 update). Nucleic Acids Res 44, W83-89.

Yu, G., Wang, L.G., Han, Y., He, Q.Y., 2012. clusterProfiler: an R package for comparing biological themes among gene clusters. OMICS 16, 284-287.
